# An Interdisciplinary Home-Based Medical Care Program to Reach Homebound Adults Living With Dementia

**DOI:** 10.1093/geroni/igab046.1797

**Published:** 2021-12-17

**Authors:** Meghan Mattos, Karen Duffy, Tuula Ranta, Justin Mutter

**Affiliations:** 1 University of Virginia, School of Nursing, Charlottesville, Virginia, United States; 2 UVA Health, Charlottesville, Virginia, United States; 3 University of Virginia, School of Medicine, Charlottesville, Virginia, United States

## Abstract

The COVID pandemic has impacted access to care, particularly for older, homebound persons living with dementia (PwD). At the beginning of the pandemic, our interdisciplinary team introduced a home-based medical care program (HBMC) to address chronically ill and homebound PwD and caregivers’ needs to promote aging in place. The purpose of this presentation is to describe PwD and caregiver service use and experiences with Virginia at Home (VaH) HBMC during the pandemic. All PwD participating in VaH program are offered home telehealth access with necessary devices. We will discuss telehealth access and use and dyad-care provider communication across up to 20 dyads to facilitate continuity of care. These data are supplemented by qualitative interviews with dyads presenting needs, preferences, and experiences accessing and using services across the first six months of program launch. We will conclude with a discussion of participant-informed program alterations to facilitate optimal overall care and outcomes.

